# The Combined Effects of Topography and Stiffness on Neuronal Differentiation and Maturation Using a Hydrogel Platform

**DOI:** 10.3390/cells12060934

**Published:** 2023-03-18

**Authors:** Sabrina Mattiassi, Abigail A. Conner, Fan Feng, Eyleen L. K. Goh, Evelyn K. F. Yim

**Affiliations:** 1 Department of Chemical Engineering, University of Waterloo, 200 University Avenue West, Waterloo, ON N2L 3G1, Canada; 2 Neuroscience and Mental Health Faculty, Lee Kong China School of Medicine, Nanyang Technological University, Singapore 308232, Singapore; 3 Waterloo Institute for Nanotechnology, University of Waterloo, 200 University Avenue West, Waterloo, ON N2L 3G1, Canada; 4 Center for Biotechnology and Bioengineering, University of Waterloo, 200 University Avenue West, Waterloo, ON N2L 3G1, Canada

**Keywords:** neuronal differentiation, mechanobiology, topography, stiffness, polyacrylamide hydrogel, mouse neural progenitor cell, human neural progenitor cell

## Abstract

Biophysical parameters such as substrate topography and stiffness have been shown independently to elicit profound effects on neuronal differentiation and maturation from neural progenitor cells (NPCs) yet have not been investigated in combination. Here, the effects of various micrograting and stiffness combinations on neuronal differentiation and maturation were investigated using a polyacrylamide and N-acryloyl-6-aminocaproic acid copolymer (PAA-ACA) hydrogel with tunable stiffness. Whole laminin was conjugated onto the PAA-ACA surface indirectly or directly to facilitate long-term mouse and human NPC-derived neuron attachment. Three micrograting dimensions (2–10 µm) were patterned onto gels with varying stiffness (6.1–110.5 kPa) to evaluate the effects of topography, stiffness, and their interaction. The results demonstrate that the extracellular matrix (ECM)-modified PAA-ACA gels support mouse and human neuronal cell attachment throughout the differentiation and maturation stages (14 and 28 days, respectively). The interaction between topography and stiffness is shown to significantly increase the proportion of β-tubulin III (TUJ1) positive neurons and microtubule associated protein-2 (MAP2) positive neurite branching and length. Thus, the effects of topography and stiffness cannot be imparted. These results provide a novel platform for neural mechanobiology studies and emphasize the utility of optimizing numerous biophysical cues for improved neuronal yield in vitro.

## 1. Introduction

The structural properties of the neural microenvironment provide biophysical cues that mediate regeneration and development in the central nervous system (CNS) [1]. Biophysical cues, namely stiffness and topography, are derived from the extracellular matrix (ECM) and can be harnessed to design biomimetic materials for neuronal differentiation in vitro [2]. A wealth of research has demonstrated the use of nano- and microtopographical patterns in enhancing neuronal differentiation [3,4,5,6,7,8,9,10,11,12,13]. Such reports emphasize the utility of anisotropic patterns such as continuous gratings, also referred to as grooves or ridges, in modulating cell behavior through the contact guidance phenomenon. The mechanosensitive response of progenitor cells on soft substrates with Young’s moduli in the range of brain tissue (0.1–1.4 kPa) [14] is well-established. Several studies have shown that substrate stiffness can drive neuronal differentiation in the absence of biochemical factors [15,16,17,18]. While topography and stiffness have been extensively studied independently, their combined effect on neuronal differentiation has not been studied systematically. The optimization of numerous biophysical parameters can be utilized to enhance neuronal differentiation and replace the current platforms that require biochemical factors. This work aims to provide and utilize a polyacrylamide (PAA) and N-acryloyl-6-aminocaproic acid (ACA) copolymer (PAA-ACA) hydrogel for studies of the combined effects of topography and stiffness on mouse and human neural progenitor cell (mNPC and hNPC, respectively) differentiation and maturation.

To study mechanosensitive lineage commitment, it is necessary to develop a platform that can undergo topographical modifications, possesses varying rigidities, and facilitates long-term cell attachment. The formation of mature neurons from NPCs may take weeks to months and requires cell attachment throughout the duration of the maturation phase. Due to their tunable stiffness and capacity to undergo micropatterning, PAA hydrogels are commonly used for mechanobiology studies. However, cellular attachment to PAA hydrogels is limited and requires coatings with ECM components. The copolymerization of PAA with ACA facilitates the use of carbodiimide crosslinking chemistry to form stable amide linkages with proteins, thus covalently tethering them to the surface [19]. Yip et al. developed a PAA-ACA copolymer that enables the stable immobilization of collagen onto hydrogel surface [20] and our group has previously developed a technique to micropattern the gels [21]. Here, we seek to adopt the approach used by Yip et al. to immobilize laminin onto the PAA-ACA surface either directly or via cationic polypeptide intermediates, thereby improving neuronal attachment.

A key barrier in neural tissue engineering is the low yield of mature neuronal cells from pluripotent progenitors, which have a propensity to differentiate into astrocytes [15]. As described in Soni et al., neurons comprise only around 30% of the cell population derived from NPCs [22]. Low-yield presents both a technical challenge for achieving biologically relevant conclusions in basic science studies and a hurdle for clinical translation and application. Furthermore, neural cells derived from pluripotent stem cells have been shown to resemble neonatal or fetal neurons rather than adult cells [23,24]. Therefore, improving the rate of neuronal maturation is also of great importance to neural tissue engineering and disease modeling, and can be gauged using transcriptomic or morphological features [24]. This research aims to determine which combinations of biophysical cues can improve the neuronal differentiation over glial differentiation and the rate of neuronal maturation using protein and mRNA detection as well as neurite arborization. We hypothesized that stiffness and topography elicit a synergistic effect on NPC differentiation and maturation into neurons.

To address the hypothesis, we first optimized the laminin coating on the PAA-ACA hydrogel surface to support mouse NPC (mNPC) and human NPC (hNPC) attachment. Secondly, we showed that the combination of topography and stiffness can be used to significantly increase mNPC neuronal differentiation based on the expression of β-tubulin III (TUJ1) relative to glial acidic fibrillary protein (GFAP) and promote neuronal maturation. Markers of neuronal differentiation (TUJ1) and maturation (microtubule associated protein 2, MAP2) were promoted by softer, more physiologically relevant hydrogels and showed a general preference towards smaller topography dimensions. Thirdly, we showed that topography and stiffness combinations can significantly enhance neurite branching and elongation. We demonstrated that a gel with a Young’s moduli of 6.1 kPa and microgratings with a height, spacing, and width of 5 µm results in the highest yield of mNPC-derived neurons with increased neurite length and branching.

## 2. Materials and Methods

### 2.1. PAA-ACA Gel Fabrication Overview

**Figure 1:**
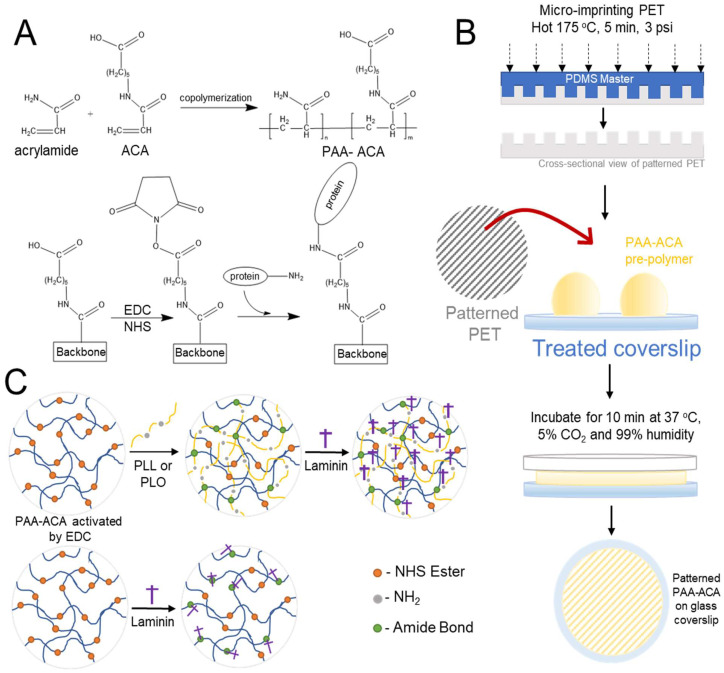
Schematic overview of the polyacrylamide (PAA)- N-acryloyl-6-aminocaproic acid (ACA) gel fabrication process and carbodiimide crosslinking chemistry: (**A**) Acrylamide and ACA are copolymerized using free radical polymerization (top), which is followed by the use of carbodiimide crosslinking chemistry to conjugate polypeptides to the copolymer backbone. (**B**) The process of patterning microtopographies on the PAA-ACA gel surface using polyethylene terephthalate (PET) molds. Hot embossing is used to microimprint patterns on PET from a PDMS master mold. The PET mold is then placed on top of the copolymer solution on the activated glass coverslips and incubated to facilitate copolymerization, resulting in a patterned PAA-ACA gel attached to a glass coverslip. (**C**) The conjugation of extracellular matrix (ECM) proteins either by direct conjugation (bottom) or by polypeptide (PLL or PLO) intermediates (top).

A summary of gel fabrication is provided in Figure 1. Briefly, a copolymer solution of solution polyacrylamide (PAA) and N-acryloyl-6-aminocaproic acid (ACA) is produced according to the protocols of Yip et al. 2013 [20] and Yip et al. 2018 [21]. Varying concentrations of acrylamide and bis-acrylamide are added to a constant concentration of ACA to produce gels of four distinct stiffnesses. The copolymer (PAA-ACA) solution is added to glass coverslips that have been activated with aminosilanes for cell attachment. A polyethylene terephthalate (PET) mold with gratings is then placed on top of the glass coverslip, sandwiching the prepolymer solution and allowing for the formation of microgratings with 1:1 aspect ratios. Laminin is either directly conjugated to the PAA-ACA gel or by a polypeptide intermediate using carbodiimide crosslinking chemistry.

### 2.2. Activation of Glass Coverslips

For silanziation, 12 mm diameter glass coverslips (1254581, Fisher Scientific, Waltham, MA, USA) were sterilized and submerged fully in a 0.5% (*v*/*v*) aqueous solution of (3-aminopropyl)triethoxysilane within 3D printed polylactic acid holders (Model ID 3DPX-012889, NIH 3D Print Exchange) at for 30 min with occasional agitation. Silanized glass coverslips are rinsed six times with deionized water and dried fully. To generate aldehyde groups, the coverslips are submerged in a 0.5% (*v*/*v*) glutaraldehyde solution (G5882, Sigma Aldrich, Munich, Germany) in 1X phosphate buffered saline (PBS) for 30 min and then rinsed three times with deionized water. Excess water is blotted away, and coverslips are allowed to dry fully at room temperature and then stored in calcium chloride at 4 °C for up to two months. All activation steps are performed at room temperature.

### 2.3. Hot Embossing of PET Molds

Master molds for hot embossing are generated from 1:10 polydimethylsiloxane (PDMS) (184 Kit 4019862, Ellsworth Adhesives Canada, Stoney Creek, ON, Canada) via soft lithography from silicon masters (Figure 1B). Molds contain gratings with the following dimensions in micrometers (height/depth × width × spacing): 2 µm × 2 µm × 2 µm, 5 µm × 5 µm × 5 µm, and 10 µm × 10 µm × 10 µm. For simplicity, these dimensions are referred to as 2 µmG, 5 µmG, and 10 µmG throughout Section 3 and represented as 2 uG, 5 uG, and 10 uG, respectively, in figures. In all cases, the aspect ratio (depth:width) of the micrograting is 1:1 to simplify the study of topography dimensions. Polyethylene terephthalate (PET) (ES301445, Goodfellow Cambridge Limited, Huntingdon, UK) molds are fabricated by conventional hot embossing. Briefly, PET is sterilized in 100% ethanol, dried, and then heated above its glass transition temperature. The PDMS mold is placed pattern down on the PET sample. A five pound weight is applied to the PDMS master and held in place for five minutes and then removed from heat. Once cooled to room temperature, PDMS master is demolded and the patterned PET is cut into the dimensions of the glass coverslip, sterilized, and air plasma treated for 30 s at 85 W and 0.8 NL/h.

### 2.4. Co-Polymerization of Polyacrylamide and N-Acryloyl-6-Aminoproic Acid and Micropatterning with PET Molds

A copolymer of polyacrylamide (PAA) and N-acryloyl-6-aminocaproic acid (ACA) (A1896, Tokyo Chemical Industry, Tokyo, Japan), referred to as PAA-ACA, was synthesized as previously described [20]. Briefly, a solution of 500 mM ACA is prepared in aqueous 0.35 M sodium hydroxide (NaOH) and filter sterilized. The prepolymer solution is formed by the combination of acrylamide (1610–0140, Bio-Rad, Hercules, CA, USA), bis-acrylamide (161–0142 Bio-Rad, Hercules, CA, USA), ACA, and deionized water in concentrations indicated in Table S1. Varying concentrations of acrylamide and bis-acrylamide are used to vary gel stiffness. Once homogeneous, aqueous tetramethyl ethylenediamine (TEMED) buffer is added to the prepolymer solution and mixed. Aqueous ammonium persulfate (A3678 Sigma Aldrich), the free radical initiator, is added and mixed. A total of 20 μL of the prepolymer solution is added to the activated coverslips in droplets in a petri dish. The PET mold is placed on top of the prepolymer, and the samples are added to an incubator at 37 °C, 5% CO_2_, and 99% humidity for 10 min to achieve full free radical polymerization. 100 mM 2-(N-morpholino)ethane sulfonic acid and 0.9% (*w*/*v*) sodium chloride (MES) buffer is prepared by dissolving the BupH MES buffered saline packs (28390 ThermoFisher Scientific) in deionized water and adjusted to pH 6.1 using 5 M NaOH. The resulting gels are immersed in 100 mM MES buffer and then stored in 100 mM MES buffer either demolded or with the PET mold at 4 °C still attached to the glass coverslip.

### 2.5. Conjugation of Polypeptides and Extracellular Matrix to the PAA-ACA Gels

Polypeptides and ECM proteins were conjugated to the PAA-ACA gel surface to promote cell adhesion. Gels are washed thrice with MES buffer and then undergo UV sterilization for 30 min. A 0.5 M N-hydroxysuccinimide (NHS) (130672, Sigma Aldrich) and 0.2 M N-(3-dimethylaminopropyl)-N’-ethylcarbodiimide hydrochloride (EDC) (221465, Sigma Aldrich) in MES buffer is prepared fresh and kept chilled until use. After sterilization, the gels are activated in excess EDC/NHS solution and then submersed in the 0.5 M NHS/0.2 M EDC solution and incubated on a rocker at room temperature for 30 min. Unreacted EDC/NHS is then removed by rinsing the samples with 1X PBS three times.

For direct conjugation of ECM, the gels are incubated with 500 μL of 20 μg/mL of laminin from murine Englebreth-Holm-Swarm (EHS) sarcoma (354232, Corning) in 1X PBS for 2 h in the incubator at 37 °C, 5% CO_2_, and 99% humidity. To visualize direct laminin conjugation, a 1:5 ratio of fluorescent HiLyte 488™ laminin (LMN02, Cytoskeleton Inc., Denver, CO, USA) and EHS sarcoma laminin was used.

For the addition of polypeptide intermediates, amide linkages are then formed by the incubation of the samples with either poly-L-ornithine hydrobromide (PLO) (MW = 30,000–70,000) (P4957, Sigma Aldrich) diluted to 0.07 mg/mL in 1X PBS or poly-L-lysine (PLL) (MW = 30,000–70,000) (P2636, Sigma Aldrich) diluted to 0.5 mg/mL in 1X PBS. The coating concentrations of PLO and PLL were 0.0175 mg/cm^2^ and 0.125 mg/cm^2^, respectively, contrasting the supplier’s recommended density of 0.002 mg/cm^2^. The higher coating concentration is owed to the assumption that the crosslinking reaction would not consume all of the PLO and PLL. Samples were incubated with 500 μL of the polypeptide solution for two hours at room temperature. The gels are rinsed with 1X PBS thrice for 15 min. The samples are then placed in solutions of either 20 μg/mL mouse EHS laminin in 1X PBS or 20 μg/mL mouse EHS laminin with 1 mg/mL heparin sulfate in 1X PBS. The latter solution was allowed to react for a minimum of 2 h at 4 °C. The gels are incubated with the ECM solutions for 2 h in the incubator at 37 °C, 5% CO_2_, and 99% humidity. Both the directly and indirectly conjugated samples are washed with 1X PBS and either stored at 4 °C for no more than a week or used immediately.

### 2.6. Mechanical Characterization and Optical Profilometry of the PAA-ACA Gels

The compressive Young’s modulus of the PAA-ACA samples was measured using a MicroTester (CellScale, Waterloo, ON, Canada). The tests are conducted in a bath of 1X PBS at 37 °C, with samples equilibrated in the bath for five minutes prior to testing. No pre-load is applied, and the sample is compressed at a rate of 5 μm/s until approximately 30% strain is achieved, after which the plate is raised an equivalent rate until reaching its starting position. Five samples of each stiffness group were used for mechanical measurements. Optical profilometry was conducted with the LEXT OLS5000 3D Laser Scanning Microscope (Olympus, Tokyo, Japan). Fully hydrated samples are used for imaging. Surface topography profiles are generated from the stiffest sample (92.6 kPa) for each topography group. Three replicates with 10–12 measurements each are used to calculate average dimensions of each topography group.

### 2.7. Neural Progenitor Cell Cutlure, Maintenance, and Differentiation

Primary mNPCs were isolated at the laboratory of the author EG in the National University of Singapore and were isolated from the hippocampal region of a day-5 postnatal mouse brain in accordance with Institutional Animal Care and Use Committee (IACUC) guidelines, using in-house protocol, which was adapted from Natalie D. Bull and Perry F. Bartlett. The protocol has been previously published in Shivaraj et al. [25]. mNPCs are expanded in mouse NPC maintenance media, the composition of which can be found in Table S2 and cultured in 6-well plates coated with natural mouse laminin (Invitrogen, 22017015). Cells are passaged once they reached 95% confluence with a split ratio of 1:2 or 1:3 and are passaged four times before use. Half of the maintenance media is changed every other day and the cells were grown in an incubator at 37 °C, 5% CO_2_, and 99% humidity. mNPCs within passages 15–19 were used for all experiments. Human fibroblasts were reprogrammed according to Chin et al. to generate induced pluripotent stem cells (iPSCs) [26]. The neural induction of the iPSCs was conducted in the lab of E.L.K.G. according to the protocols of Chin et al. [26] and Su et al. [27]; the resulted hNPCs obtained in Chin et al. are used in this study. All work with human cells is performed under the University of Waterloo Ethics Approval (41244). hNPCs are maintained in human NPC maintenance media, the composition of which can be found in Table S3. hNPCs were cultured in 6-well plates coated with Matrigel^®^ Basement Membrane Matrix, LDEV-free (354248, Corning, Corning, NY, USA) until reaching 90% confluence. After passaging, cells are seeded with the p160-Rho-associated kinase (ROCK) inhibitor 0.5 μM 4-[(1R)-1-aminoethyl]-N-4-pyidinyl-transcyclohexanecarboxamide (Y-27632) (Y-27632, STEMCELL Technologies, Vancouver, Canada). Half of the media is changed every 48 h and hNPCs grown in an incubator at 37 °C, 5% CO_2_, and 99% humidity. hNPCs within passages 30–48 were used for all experiments.

mNPCs are differentiated by switching from the maintenance media to the mNPC induction media, the composition of which can be found in Table S2. Half of the media is changed every 48 h and cells are maintained in the mNPC induction media for 7 days. Subsequently, cells are cultured in the mNPC maturation media (Table S2). Half of the maturation media was changed every 48 h and mNPCs were maintained in the maturation media for 7 days. Similarly, hNPCs are differentiated by switching to the hNPC differentiation media, the composition of which is shown in Table S3. The mNPC differentiation period lasted 14 days whereas the hNPC differentiation period lasted 21 days for RNA extraction and 28 days for confirmation of cell adhesion, during which both cell types were kept in an incubator at 37 °C, 5% CO_2_, and 99% humidity.

### 2.8. Cell Seeding on PAA-ACA Hydrogels

Prior to use, gels are washed and allowed to soak in DMEM/F12 for 30 min in the incubator. To confirm adhesion to the hydrogel surface, mNPCs were seeded at 20,000 cells/cm^2^ in mNPC maintenance media. hNPCs were seeded at 20,000 and 5000 cells/cm^2^ and supplemented with 5 μM ROCK inhibitor Y-27632. After 24 h, phase-contrast microscopy was used to confirm adhesion, and, if adherent, the mNPC and hNPC maintenance media was switched to the mNPC induction and hNPC induction media to begin differentiation.

### 2.9. Immunofluoresence Staining and Imaging

After 14 days and 21/28 days, the mNPCs and hNPCs, respectively, were fixed for immunofluorescence staining and imaging. Fixation was achieved using 4% paraformaldehyde, followed by permeabilization with 0.25% Triton X-100 (T8787, Sigma Aldrich) and blocking with 1% (*w*/*v*) bovine serum albumin or 10% (*v*/*v*) goat serum in 1X tris-buffered saline (TBS, Atlanta, GA, USA) (pH 7.4) (Bp2471-500 Fisher BioReagents). Following blocking, cells were immunostained with β-tubulin III (TUJ1) (rabbit anti-TUJ1 at 1:600 polyclonal, Sigma Aldrich), glial acidic fibrillary protein (GFAP) (mouse anti-GFAP at 1:600, polyclonal, Sigma Aldrich), or microtubule associated protein 2 (MAP2) (mouse anti-microtubule associated protein at 1:600, polyclonal, Abcam) overnight at 4 °C. Samples were then washed thrice with 0.05% Triton X-100 and 1% goat serum in 1X TBS. Alexa Fluor 488 goat anti-rabbit IgG (A11034 Invitrogen™, Waltham, MA, USA) and Alexa Fluor 546 goat anti-mouse IgG (A11003 Invitrogen™) in 1X TBS were used as secondary antibodies for TUJ1 and MAP2, respectively. Secondary immunostaining was carried out overnight at 4 °C. Samples were counterstained with 4′,6-Diamidino-2-Phenylindole, Dihydrochloride (DAPI) solution (1:2200) (D9542 Sigma Aldrich) for 1 h at room temperature and then mounted on glass coverslips for fluorescence microscope (Axio Observer Z1, Zeiss GmBH) analysis. Fluorescent microscope images were analyzed with the ImageJ and Cell Profiler software. The ImageJ plugin NeuriteJ was used for measuring the neurite length.

### 2.10. RNA Isolation and RT-qPCR

After 21 days, total RNA was isolated from hNPCs cultured on the PAA-ACA hydrogels using the QIAGEN RNeasy Mini Kit (74104, QIAGEN, Hilden, Germany). Three biological replicates were used to harvest total RNA. The quantity and quality of the total RNA samples was determined using the NanoDrop ND-1000 Spectrophotometer. The total RNA samples were stored at −80 °C. The iScript™ cDNA Synthesis Kit was used for reverse transcription (RT) (170-8891, Bio-Rad, Hercules, CA, USA)of the total RNA. cDNA samples were quantified using the NanoDrop ND-1000 Spectrophotometer, diluted in DPEC-treated RNase free DI H_2_O into aliquots, and stored at −20 °C until use in qPCR. All cDNA samples had a 260/280 ratio of greater than 1.80. qPCR was used to determine relative expression levels of human *MAP2* using the TaqMan^TM^ Gene Expression Assays (Applied Biosystems, 4331181) for *MAP2* (Assay ID: Hs00258900_m1) and *GAPDH* (Assay ID: Hs02786624_g1), the latter serving as the endogenous control. TaqMan Advanced Fast Mastermix and DPEC-treated nuclease free DI H_2_O were used to prepare the reaction mixture. Applied Biosystems Two technical replicates of each sample were on the plate, and 2 technical replicates of each plate were used. Relative *MAP2* expression was determined via the $2^{-\Delta \Delta C_{T}}$ (Livak-Schmittgen) method, normalizing to glyceraldehyde-3-phosphate dehydrogenase (GAPDH) expression on the coverslip control.

### 2.11. Statistics

Statistical analysis was performed in GraphPad Prism 9. A two-way ANOVA and Turkey’s post-hoc test with an alpha of 0.05 was used to determine statistical significance. For all analyses, the cells grown on the glass coverslip were used as the control and were not included in the ANOVA or in the pairwise analysis. All values are reported as a mean with a 95% confidence interval. The symbols *, **, ***, ****, and n.s. represent *p* < 0.05, *p* < 0.01, *p* < 0.001, *p* < 0.0001, and *p* > 0.05 (not significant), respectively. Spearman’s coefficient, r, was used to assess the strength and direction of the correlation between factors. Correlation coefficients between 0.1–0.29, 0.3–0.49, and greater than 0.5 were considered to be low, moderate, and high, respectively. For correlation analysis, topography was treated as a continuous variable, with the blank sample given a value of 100,000 (treated as infinitely large topography). The number of biological and technical replicates for the immunofluorescence imaging analysis is denoted in the respective figure legends. Figures containing graphs or a heatmap are generated in GraphPad Prism 9. 

## 3. Results

### 3.1. Physical Characterization of the PAA-ACA Gels

**Figure 2:**
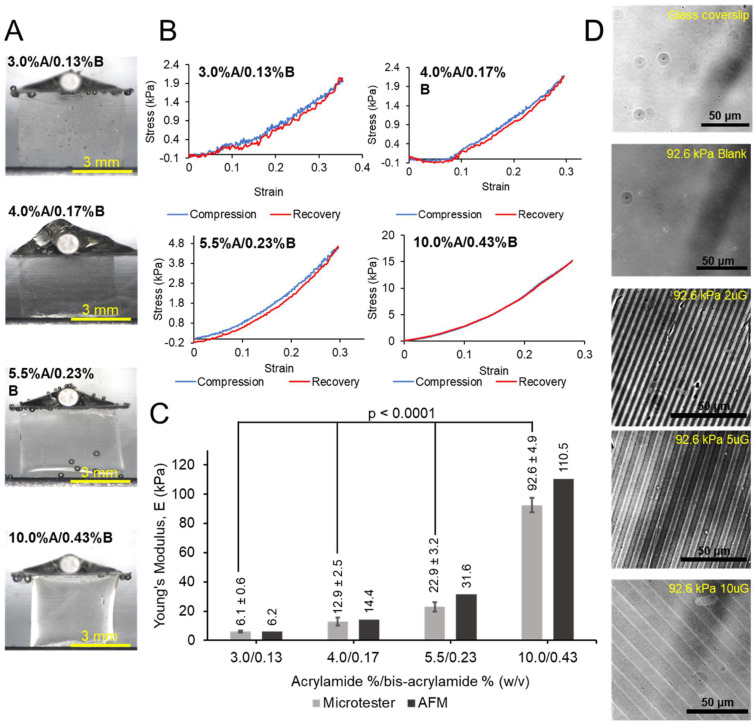
Physical characterization of the PAA-ACA gels: (**A**) Representative images of the starting conditions for the mechanical testing of the hydrogel samples. (**B**) Stress versus strain curves for samples of varying ratios of acrylamide to bis-acrylamide. (**C**) Measurements of the Young’s modulus as determined by the MicroTester compared to the AFM measurements completed by Yip et al. [21]. The Young’s moduli measurements are used to classify the stiffness groups throughout this work. (**D**) Representative images of micropatterns (2 uG = 2 µmG; 5 uG = 5 µmG; 10 uG = 10 µmG) from the stiffest PAA-ACA gel group. The blank sample has no topographical modifications, and the glass coverslip is used as a control.

The process of fabricating the PAA-ACA gels is summarized in Figure 1. The resulting gels were completely transparent, had an average height of 196.20 ± 13.86 μm, and remained attached to the glass coverslips throughout the duration of cell culture. The results of the compressive uniaxial tests and optical profilometry are shown in Figure 2. The various ratios (*w*/*v*) of acrylamide (A) to bis-acrylamide (B) are denoted as %A/%B in Figure 2 and in the text herein. The average Young’s moduli of the gels in 1X PBS at 37 °C with acrylamide to bis-acrylamide ratios of 3.0%A/0.13%B, 4.0%A/0.17%B, 5.5%A/0.23%B, and 10%A/0.43%B were found to be 6.1 ± 0.6 kPa, 12.9 ± 2.5 kPa, 22.9 ± 3.2 kPa, and 92.6 ± 4.9 kPa, respectively (reported as 95% CI) (Figure 2C). Furthermore, the Young’s Moduli were shown to be significantly different from each other (*p*-value = 3.04 × 10^−16^) (Figure 2C). As shown in Figure 2B, the stress-strain curves of all stiffness groups are reminiscent of soft tissue. Furthermore, the gels are purely elastic, as indicated by relationship between the compression and recovery curves (Figure 2B). Figure 2D shows the presence of microtopography on the surface of the stiffest gel (96.2 kPa), demonstrating the success of micropatterning. Using a method similar to our previous study [21], gratings of three different dimensions were imparted on the gel surface (depth × width × spacing in μm): 2 μm × 2 μm × 2 μm (2 µmG), 5 × 5 × 5 (5 µmG), and 10 × 10 × 10 (10 µmG). The dimensions for the 2 µmG pattern were determined to be 2.03 ± 0.12 μm height × 1.85 ± 0.19 μm width × 2.13 ± 0.15 μm spacing. The dimensions for the 5 µmG pattern were determined to be 5.64 ± 0.21 μm height × 5.31 ± 0.27 μm width × 5.02 ± 0.28 μm spacing. The dimensions for the 10 µmG pattern were determined to be 10.05 ± 0.65 μm height × 10.21 ± 0.40 μm width × 10.51 ± 0.49 μm spacing.

### 3.2. Extended Attachment of mNPCs and hNPCs to the ECM-Coated PAA-ACA Gels

**Figure 3:**
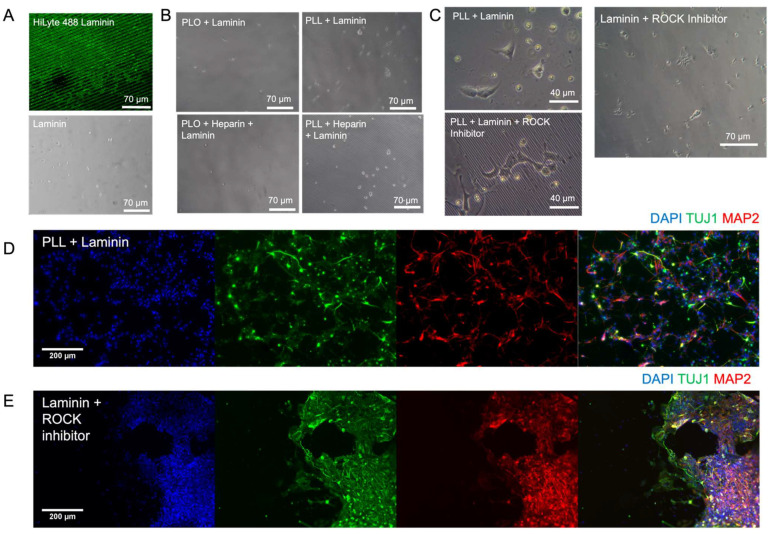
The attachment, differentiation, and maturation of mNPCs and hNPCs on the ECM-conjugated PAA-ACA gels: (**A**) Confirmation of direct laminin conjugation as visualized by the green, fluorescent HiLyte laminin (top) and mNPC attachment to the directly conjugated laminin ECM coating (bottom). (**B**) The attachment of mNPCs to laminin and laminin-heparin solution conjugated to the gel surface via two cationic polypeptide intermediates: PLO (left) and PLL (right). (**C**) The attachment of hNPCs to PLL-conjugated laminin substrates (left) and direct laminin conjugated gels (right), in the absence and presence of ROCK inhibitor. (**D**) mNPC-derived neurons on blank PAA-ACA substrates with PLL and laminin ECM coating fixed after a differentiation period of 14 days and stained with TUJ1 and MAP2 antibodies. Counterstained with DAPI. (**E**) hNPC-derived neurons on blank PAA-ACA substrates with direct laminin-conjugation and the addition of ROCK inhibitor, fixed after a differentiation period of 28 days and stained with TUJ1 and MAP2 antibodies. Counterstained with DAPI.

To improve neuronal attachment to the PAA-ACA gels, laminin was conjugated to the surface either directly or by a cationic polypeptide intermediate. The concentration of ACA was constant for all gels to ensure there was a consistent and even distribution of conjugated cell attachment factors. Figure 3A (top) provides confirmation of the direct conjugation of laminin to the PAA-ACA gel via carbodiimide cross-linking chemistry. As shown in Figure 3A (bottom), mNPC attachment to the directly-conjugated laminin is poor. Similarly, mNPC attachment to the PLO and laminin as well as the PLO and laminin-heparin solutions was minimal, and the cells expressed a round morphology (Figure 3B). When the laminin-heparin solution was used as the ECM coating for the PLL conjugated samples, there was also limited mNPC attachment. Contrastingly, laminin-coated samples with PLL intermediate showed strong cell attachment, with most cells possessing an elongated morphology indicative of healthy mNPCs. The hNPCs showed minimal attachment to the PAA-ACA substrates without the addition of Y-27632, which is an inhibitor of p160-Rho-associated kinase (ROCK) (Figure 3C). However, directly conjugated laminin without the use of a polypeptide intermediate was sufficient to facilitate hNPC attachment, as demonstrated by the number of elongated cells on this substrate. For subsequent studies, mNPCs were cultured on gels with the PLL-conjugated laminin and hNPCs were cultured on gels with directly conjugated laminin with the addition of ROCK inhibitor directly after seeding. On these substrates, the mNPCs and hNPCs were capable of differentiating into neurons, as demonstrated by the expression of the class III β-tubulin (TUJ1) in Figure 3C,D, respectively. TUJ1 is a neuron-specific tubulin that arises early in the commitment to the neuronal lineage and is thus a common marker of neuronal differentiation [28]. Furthermore, as indicated by the expression of microtubule associated protein 2 (MAP2) in Figure 3C,D the mNPCs and hNPCs, respectively, underwent neuronal maturation. MAP2 expression is confined to the dendrites of post-mitotic, terminally differentiated neurons and is thus frequently used as a marker of neuronal maturation [29]. In summary, these results show that the PAA-ACA substrates with the conjugation of whole laminin facilitates the long-term attachment, differentiation, and maturation of both murine and human NPCs.

### 3.3. The Effect of Topography and Stiffness on mNPC Commitment to the Neuronal Lineage

**Figure 4:**
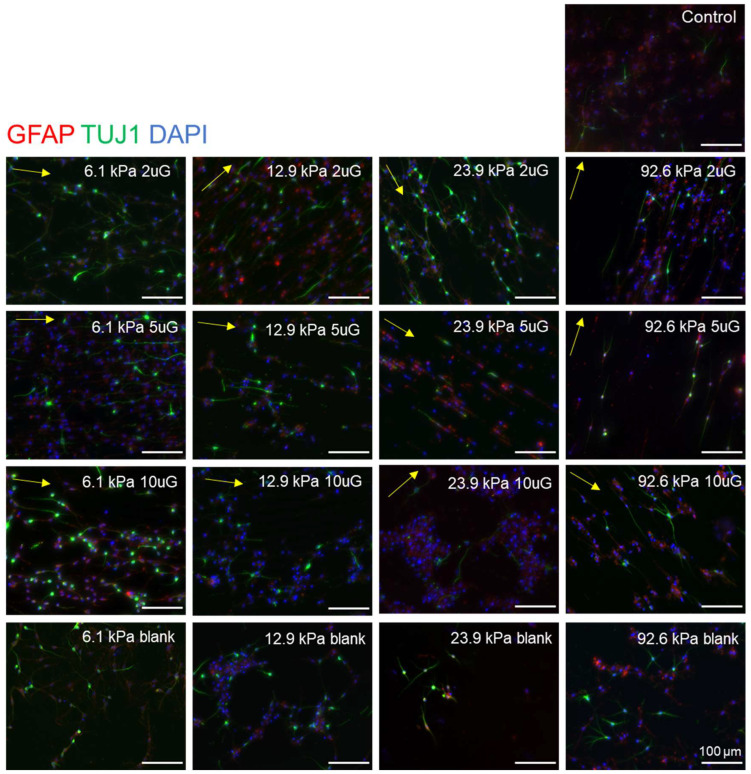
Representative images of mNPC TUJ1 and GFAP expression on PAA-ACA gels of varying stiffness and topography after a differentiation period of 14 days. mNPC-derived neurons were fixed after 14 days and then stained with TUJ1 and GFAP antibodies and counterstained for DAPI. The Young’s modulus (stiffness group) and topography (2 uG = 2 µmG; 5 uG = 5 µmG; 10 uG = 10 µmG) is indicated at the top right corner of the image. The direction of the gratings is indicated by the yellow arrow. The control was a glass coverslip. Blank samples indicate those without a grating pattern. All scale bars represent 100 μm. For TUJ1 expression, the number of biological replicates, n = 5 with two to three technical replicates each. A total of 2000 to 7000 cells were analyzed per each stiffness and topography combination. For GFAP expression, the number of biological replicates n = 2 with two technical replicates each (except in the case of the 22.9 kPa 10 µmG group, where n = 1). A total of 110 to 1000 cells were analyzed for each stiffness and topography combination.

To determine the propensity of mNPCs to commit to the neuronal lineage over the glial lineage on PAA-ACA gels with different stiffnesses and topographies, the expression of TUJ1 and glial fibrillary acidic protein (GFAP) was evaluated. GFAP is expressed in the astrocytes of the CNS and is thus frequently used as an astrocytic marker [30]. Representative images of the expression of these markers on each stiffness and pattern combination are shown in Figure 4. Both markers are expressed on the glass coverslip control, indicating that the cells are healthy and capable of differentiation. The percentage of TUJ1 positive (TUJ1+) and GFAP positive (GFAP+) cells present on the stiffness and topography combinations are detailed in Table S4. In general, the gels with patterns had a greater percentage of TUJ1+ cells and a lower percentage of GFAP+ cells compared to the glass coverslip control. Overall, the highest percentage of TUJ1+ cells (37%) was found on the 6.1 kPa with the 5 µmG pattern and the highest percentage of GFAP+ cells (25%) was found on the unpatterned 92.6 kPa gel.

**Figure 5:**
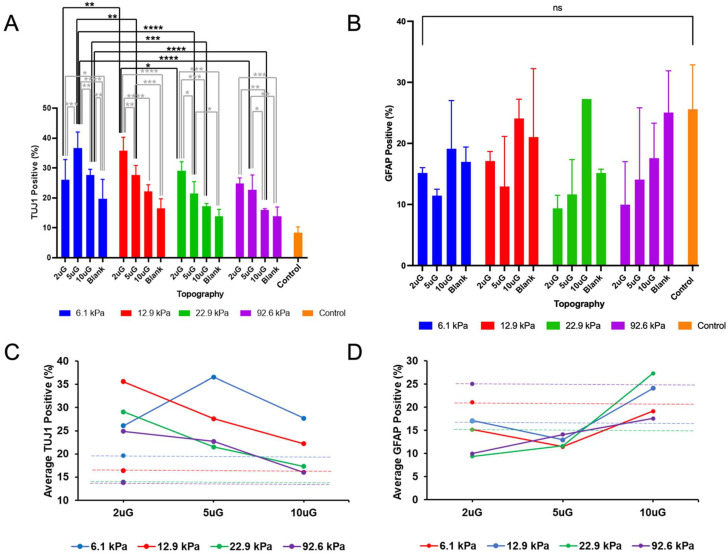
The percentage of mNPC-derived TUJ1+ cells and GFAP+ cells on PAA-ACA substrates with varying stiffnesses and gratings with varying dimensions: (**A**) Pair-wise comparisons of the percentage of TUJ1+ cells performed between and within the stiffness and topography groups. (**B**) Pair-wise comparisons of the percentage of GFAP+ cells performed between and within the stiffness and topography group. (**C**) Interaction plot of the percentage of TUJ1+ cells, topography, and stiffness. (**D**) Interaction plot of the percentage of GFAP+ cells, topography, and stiffness. For (**A**,**B**), error bars indicate the SD and the symbols *, **, ***, ****, and n.s. represent *p* < 0.05, *p* < 0.01, *p* < 0.001, *p* < 0.0001, and no significance, respectively. For (**A**,**C**), values are shown as the average of n = 5 biological replicates. For (**B**,**D**), values are shown as the average of n = 2 biological replicates (except in the case of the 22.9 kPa 10 µmG group, where n = 1).

**Figure 6:**
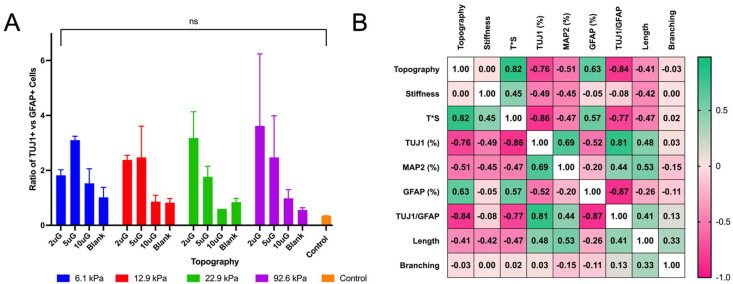
The relationship between topography, stiffness, and their interaction with markers of neuronal differentiation and maturation, neuronal and astrocyte lineage commitment, and mature neuronal morphological features: (**A**) The ratio of the total number of TUJ1+ cells to the total number of GFAP+ cells on varying stiffnesses and topographies. (**B**) Heat map of the Spearman correlation coefficients between the topography, stiffness, their interaction (T*S), neurite length, neurite branching, the ratio of the total number of TUJ1+ cells to the total number of GFAP+ cells (TUJ1/GFAP), and the percentage of TUJ1+, GFAP+, and MAP2+ cells. The direction and strength of the correlation is indicated by the color, with pink indicating negative correlation and green indicating positive correlation. For (**A**), n.s. indicates no significance or *p* > 0.05.

To perform the ANOVA analysis, topography was treated as a continuous variable with the blank sample considered as infinitely large (100,000 µm). ANOVA analysis indicated that the topography, stiffness, and their combined interaction (T*S) had a significant effect (*p* < 0.0001 in each case) on the percentage of TUJ1+ cells (Figure 5A). Contrastingly, only topography had a significant effect (*p* = 0.02) on the percentage of GFAP+ cells (Figure 5B). Increasing topography dimensions had strong negative correlation (r = −0.76) (Figure 6B) with the percentage of TUJ1+ cells. A positive curvilinear relationship between topography and the percentage of TUJ1+ cells was observed, shown in the interaction plot in Figure 5C. The maxima of these relations varied in response to the stiffness: in the 12.9 kPa, 22.9 kPa, and 92.6 kPa stiffness groups, the 2 µmG patterns resulted in the maxima. In the case of the 6.1 kPa group the maximum found at the 5 µmG topography. Notably, in all stiffness groups, the 5 µmG pattern resulted in a significantly higher percentage of TUJ1+ cells compared to the unpatterned samples. Stiffness had a moderate negative correlation (r = −0.49) (Figure 6B) with the percentage of TUJ1+ cells. The parabolic curves present in Figure 5C shifted downwards as the stiffness increased. For all gels, the percentage of TUJ1+ cells decreased as stiffness increased, with the exception of the 2 µmG pattern on the 6.1 kPa gel. T*S had a strong negative correlation with the percentage of TUJ1+ cells (r = −0.86) (Figure 6B), implying that as the T*S increases the expression of TUJ1 will decrease. Topography also had a high positive correlation with GFAP expression (r = +0.63) (Figure 6B). Stiffness did not correlate with the percentage of GFAP+ cells (r = −0.05) (Figure 6B). As evidenced in Figure 5C, no significant pairwise comparisons could be made between stiffness or topography groups. While the relationship between topography and the percentage of GFAP+ cells was also shown to be curvilinear, a negative relationship for all stiffnesses was observed in contrast to the positive relationship seen in the case of TUJ1+ cells (Figure 5D). However, the stiffness did not noticeably shift the curves upwards or downwards. While no significant relationship between topography and stiffness alone could be drawn, T*S had a strong positive correlation (r = +0.57) (Figure 6B) with the percentage of GFAP+ cells.

The relative propensity of the mNPCs to commit to the neuronal lineage can be further assessed by the ratio of the total number of TUJ1+ cells to the total number of the GFAP+ cells (TUJ1/GFAP) (Figure 6A), or rather the ratio of neuronal lineage commitment to astrocyte lineage commitment. ANOVA analysis showed that topography alone had a significant effect on the TUJ1/GFAP ratio (*p* = 0.0011). Topography and T*S had a strong negative correlation (r = −0.84 and r = −0.77, respectively) with the TUJ1/GFAP ratio, yet the stiffness did not correlate (r = −0.08) (Figure 6B). Within each stiffness group, the TUJ1/GFAP ratio was highest on the 2 µmG and 5 µmG patterns.

### 3.4. The Effect of Topography and Stiffness on mNPC Maturation

**Figure 7:**
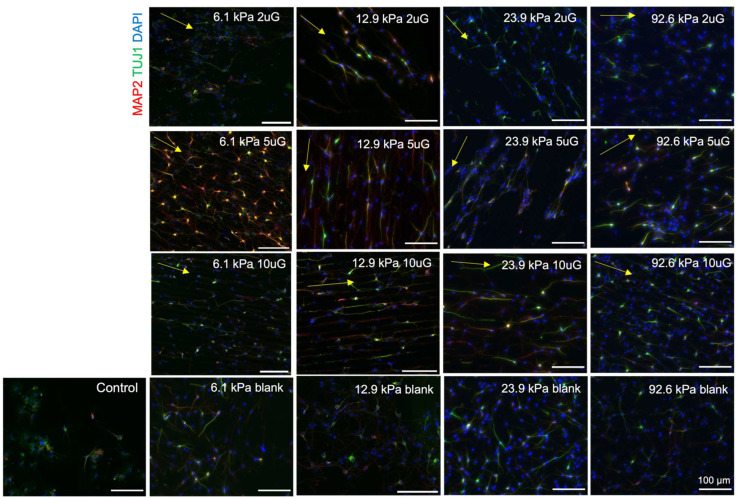
Representative images of mNPC TUJ1 and MAP2 expression on PAA-ACA gels of varying stiffness and topography after a differentiation period of 14 days. mNPC-derived neurons were fixed after 14 days and then stained with TUJ1 and MAP2 antibodies and counterstained for DAPI. The Young’s modulus (stiffness group) and topography is indicated at the top right corner of the image (2 uG = 2 µmG; 5 uG = 5 µmG; 10 uG = 10 µmG). The direction of the gratings is indicated by the yellow arrow. The control is a glass coverslip. Blank samples indicate those without a grating pattern. All scale bars represent 100 μm. For TUJ1 expression, the number of biological replicates, n = 5 with 2–3 technical replicates each. A total of 2000 to 7000 cells were analyzed per each stiffness and topography combination. For MAP2 expression, the number of biological replicates n = 3 with 3 technical replicates each. A total of 2000 to 6000 cells were analyzed per each stiffness and topography combination.

**Figure 8:**
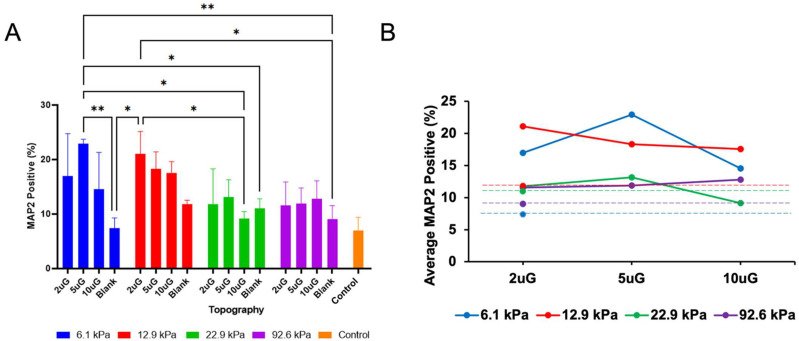
The percentage of mNPC-derived MAP2+ cells PAA-ACA substrates with varying stiffnesses and gratings with varying dimensions: (**A**) Pair-wise comparisons of the percentage of MAP2+ cells performed between and within the stiffness and topography groups. (**B**) Interaction plot of the percentage of MAP2+ cells, topography, and stiffness. For (**A**), error bars indicate the SD and the symbols *, **, and n.s represent *p* < 0.05, *p* < 0.01, and no significance, respectively. For (**A**,**B**) values are shown as the average of n = 3 biological replicates. For (**B**) data is shown as the average ±95% CI.

MAP2, a marker of mature neurons, was used to determine the extent of the neuronal maturation of mNPC derived neurons PAA-ACA substrates on varying stiffnesses and grating dimensions (Figure 7). All gels, regardless of topography or stiffness presented a higher percentage of MAP2-positive (MAP2+) cells compared to the glass coverslip control. The average percentage of MAP2+ cells can be found in Table S4. Notably, as was the case with the percentage of TUJ1+ cells, the highest percentage of MAP2+ cells was found on the 6.1 kPa gel with the 5 µmG pattern. ANOVA analysis indicated that both topography and stiffness alone (*p* = 0.0011 and *p* = 0.0009, respectively), but not T*S (*p* = 0.1), had a significant effect on the percentage of MAP2+ cells (Figure 8A). Topography had a strong negative correlation (r = −0.51) with the percentage of MAP2+ cells and stiffness had a moderate negative correlation (r = −0.45) (Figure 6B). However, no significant differences between gels of different stiffnesses with the same pattern could be drawn, as is made clear by the pairwise comparisons in Figure 8A. Despite this, there is a general trend in which the percentage of MAP2+ cells decreases as the stiffness increases. Within stiffness groups, the only significant difference occurred between the blank and the 5 µmG pattern on the 6.1 kPa gels (*p* = 0.0025) (Figure 8A). As shown in the interaction plot in Figure 8B, a curvilinear relationship between MAP2+ and topography can be observed, and the maxima of this relationship varied depending on the stiffness. The interaction plot presents a nearly linear relationship for the stiffer gels (22.9 kPa and 92.6 kPa). Despite the results of the ANOVA analysis, T*S had a moderate negative correlation (r = −0.47) with the percentage of MAP2+ cells (Figure 6B). As shown in Figure 8B, the relation between topography and percentage of MAP2+ cells becomes weakened as the stiffness is increased, flattening the curves.

### 3.5. The Effect of Topography and Stiffness on mNPC Morphology: Neurite Length and Branching

**Figure 9:**
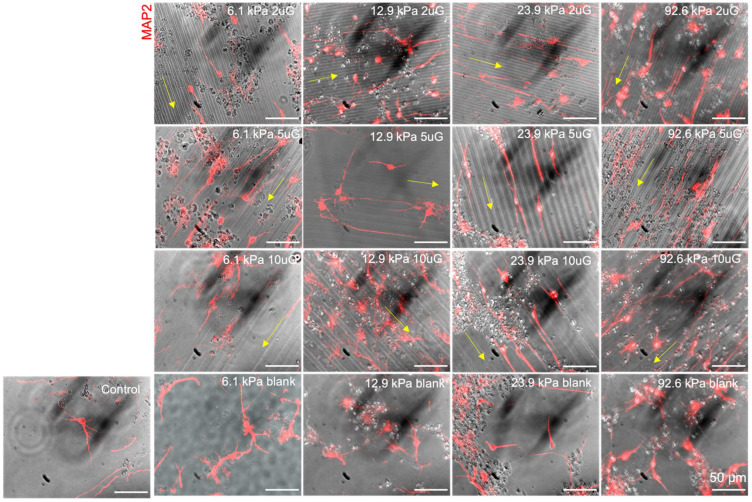
Representative images of hNPC-derived neuron MAP2+ neurites on gels with varying stiffnesses and topographies of varying dimensions, from which the neurite length, number of neurite branches, and orientation of neurites were determined. MAP2+ neurites are shown in red. The orientation of the topographies is indicated with the yellow arrow. The stiffness and topography combinations are displayed in the right corner of each panel (2 uG = 2 µmG; 5 uG = 5 µmG; 10 uG = 10 µmG). The blank sample refers to those with no pattern and the control is a glass coverslip. All scale bars represent 50 μm. The number of biological replicates n = 3.

**Figure 10:**
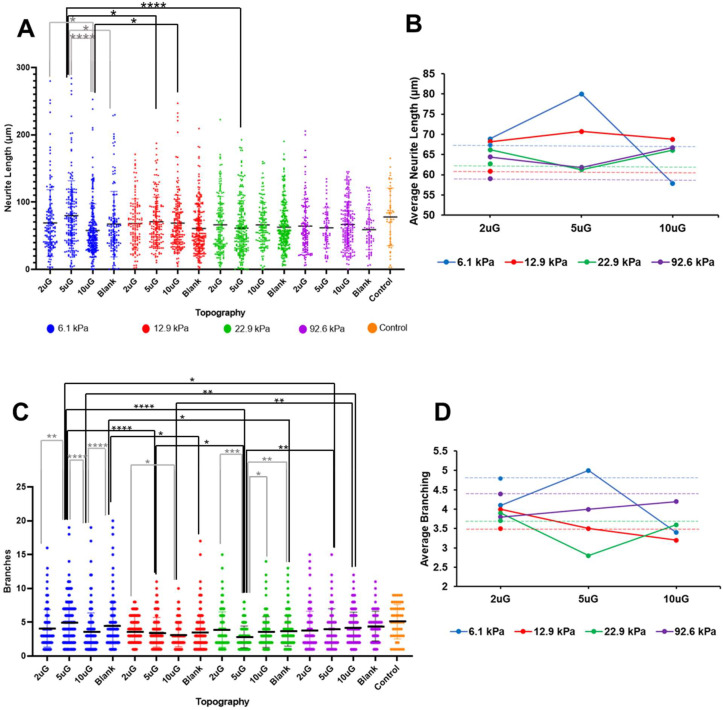
The average MAP2+ neurite length and average number of MAP2+ neurite branches of mNPCs-derived neurons grown on PAA-ACA gels of varying stiffnesses and topography dimensions: (**A**) Pair-wise comparisons of the MAP2+ neurite length in μm performed between and within the stiffness and topography groups. (**B**) Interaction plot between the average MAP2+ neurite length in μm, topography, and stiffness. (**C**) Pair-wise comparisons of the number of MAP2+ neurite branches performed between and within stiffness and topography groups. (**D**) Interaction plot between the average number of MAP2+ neurite branches, stiffness, and topography. For (**A**,**C**) error bars indicate the SD and the symbols *, **, ***, and **** represent *p* < 0.05, *p* < 0.01, *p* < 0.001, *p* < 0.0001, respectively. For (**A**–**D**) data is collected from n = 3 biological replicates. For (**B**,**D**) the data is shown as the average ±95% CI.

Neurite length and the number of neurite branches were quantified to assess neuronal morphology and thus neuronal maturation. Representative images of MAP2+ neurites are shown in Figure 9 and the average MAP2+ neurite length and the number of MAP2+ branches on different stiffness and topography combinations are presented in Table S4. On the patterned gels the neurites oriented in the direction of the gratings but grew in random directions on unpatterned substrates (Figure 9). The second longest average neurite length and the highest average rate of neurite branching was observed on the glass coverslip control. However, the longest average neurite length was observed on the 5 µmG pattern on the 6.2 kPa gel. In the 12.9 and 22.9 kPa stiffness groups, the highest average rate of neurite branching was found on the 2 µmG patterns, in contrast to the 6.2 kPa group, whereas 5 µmG pattern yielded the greatest branching. ANOVA analysis showed that only T*S had a significant effect on neurite length (*p* = 0.0002) (Figure 10A). Topography and stiffness alone did not have a significant effect (*p* > 0.07). Topography, stiffness, and their interaction had a significant effect (*p* < 0.0001, *p* = 0.0053 and *p* < 0.0001, respectively) on the average number of MAP2+ neurite branches.

Topography had a moderate negative correlation with neurite length (r = −0.41) (Figure 6B) and presented a positive curvilinear relationship for the two softer gels and a linear relationship for the stiffer gels (Figure 10B). There were no significant pairwise comparisons within stiffness groups except for the 6.2 kPa group. Here, the 2 µmG and 5 µmG patterns resulted in a significantly higher average neurite length than the 10 µmG pattern. In the other stiffness groups, the average neurite length tended to decrease as the topography dimensions increased, but no significant relations could be drawn. Stiffness had a moderate negative correlation (r = −0.42) (Figure 6B), and, in the case of the blank, 2 µmG, and 5 µmG groups the average neurite length decreased as stiffness increased (Figure 10A,B). T*S had a moderate negative correlation with the average neurite length (r = −0.47).

Topography did not correlate with the number of MAP2+ neurite branches (r = −0.03) (Figure 6B). Within all stiffness groups except for the 92.6 kPa group, significant differences between topographies was observed (Figure 10C). Neither stiffness nor T*S could be correlated with neurite branching, despite the ANOVA indicating both factors had a significant effect. However, interaction plot in Figure 10D demonstrates a clear interaction between topography and stiffness, as indicated by the numerous intersections and the lack of linearity.

### 3.6. The Effect of Topography and Stiffness on hNPC Maturation

**Figure 11:**
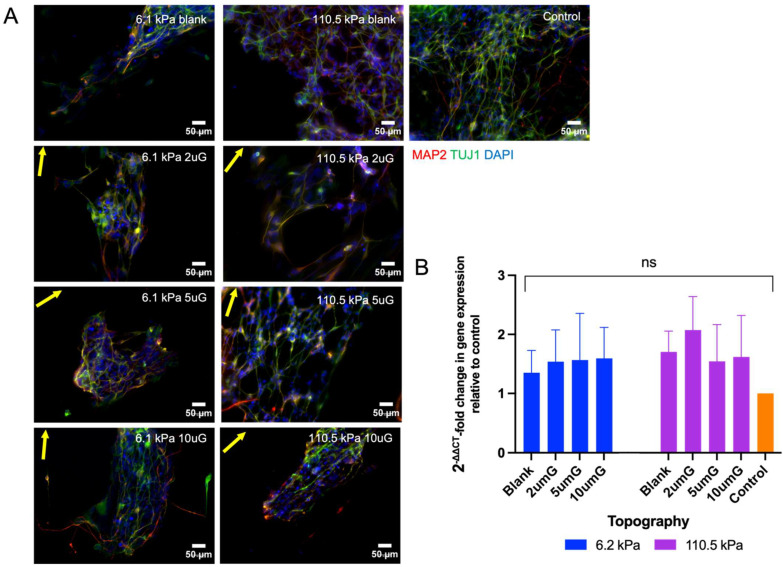
hNPC-derived neuron TUJ1 and MAP2 expression on PAA-ACA gels of varying stiffness and topography after a differentiation period of 21 days: (**A**) Representative images of hNPCs fixed and stained for TUJ1 and MAP2 and counterstained for DAPI after 21 days. The Young’s modulus (stiffness group) and topography (2 uG = 2 µmG; 5 uG = 5 µmG; 10 uG = 10 µmG) is indicated at the top right corner of the image. The direction of the gratings is indicated by the yellow arrow. The control is a glass coverslip. Blank samples indicate those without a grating pattern. All scale bars represent 50 μm. (**B**) Relative mRNA expression of MAP2 on PAA-ACA gels with stiffnesses of 6.2 kPa or 110.5 kPa and variable topographies as determined by $2^{-\Delta \Delta C_{T}}$ (Livak-Schmittgen) method and normalized to the glass coverslip control. GAPDH is the endogenous control. ns: no significance.

hNPCs were cultured on PAA-ACA gels with direct laminin-conjugated and the addition of ROCK inhibitor. Here, only the softest (6.2 kPa) and stiffest (110.5 kPa) PAA-ACA gels were selected to be studied. After 21 days, the hNPCs were fixed and stained or used for RNA extraction and RT-qPCR analysis. Representative images of the hNPC derived TUJ1+ and MAP2+ neurons are shown in Figure 11A. hNPCs were capable of maturation and differentiation on all gels. Changes in MAP2 mRNA expression on different topography and stiffness combinations relative to the glass coverslip control are evaluated in Figure 11B. The relative expression of MAP2 is higher on all gels regardless of stiffness and topography compared to that on the glass coverslip. While no significant differences could be drawn neither within nor between stiffness groups, it is clear the effect of the topography on MAP2 expression is greater within the 110.5 kPa group. In this stiffness group, MAP2 expression was highest on the 2 µmG pattern and then further declined as topography dimensions increased. Within the 6.2 kPa group, MAP2 expression is the highest on the blank, yet the difference between the patterned groups is minimal.

## 4. Discussion

A key challenge in neural tissue engineering is developing platforms that more closely mimic the native ECM to drive the production of neurons from pluripotent progenitor cells in vitro. Biophysical cues such as substrate topography and stiffness elicit profound effects on NPC cell fate and identity. Anisotropic topographies such as continuous gratings mediate neuronal cell differentiation, alignment, elongation, and behavior [3,4,5,6,7,8,9,10,11,12,13] through a phenomena known as contact guidance [31]. Brain tissue is ultrasoft [14], which presents a unique challenge in designing biomaterials that mimic its stiffness [32]. Past research has shown that materials with Young’s moduli in the range of brain tissue (0.1–1.4 kPa) [14] are sufficient to drive neuronal differentiation [15,16,17,18]. While stiffness and topography have been investigated extensively with regards to neuronal behavior, they have rarely been studied in combination. Previous works have studied the influence of both topography and stiffness on cell shape and identity [33,34,35], emphasizing the synergistic effect of multiple biophysical parameters. However, the interaction between topography and stiffness effects have not been considered with regards to neuronal differentiation. The objectives of this research seek to address this gap. Herein we have shown that indirectly or directly conjugated laminin on the PAA-ACA copolymers facilitates the attachment of mNPCs and hNPCs, respectively, throughout the direction of the induction and maturation periods (14 and 28 days, respectively). Furthermore, it has been shown that the interaction between topography and stiffness can significantly enhance the propensity of mNPCs to commit to the neuronal lineage and increase the rate of neuronal maturation. These results demonstrate the utility of biophysical cues in driving neuronal differentiation and maturation in the absence of biochemical factors. The platform developed here allows for the further optimization of stiffnesses and topographies of various dimensions and patterns to drive neuron yield.

The first aim of this work is to adopt the platform developed by Yip et al. 2013 [20] and our group [21] for neural mechanobiology studies by facilitating long-term cell attachment. The authors have developed a PAA-ACA hydrogel with tunable stiffness that is straightforward to fabricate and is optimally suited for immunofluorescent studies due to its transparency, thinness, and attachment to the glass coverslip. Importantly, as we have reaffirmed, the hydrogel stiffness can be reliably tuned by varying ratios of acrylamide to bis-acrylamide and a range of microtopographies with consistent dimensions can be patterned using adaptable PET molds. This facilitates the exploration of other stiffness and topography combinations, including isotropic topographies as well. While this platform is apt for mechanobiology studies in general, it is limited in its application to neural biology due to the requirement of long-term NPC-derived neuronal attachment. Neurons must remain attached throughout the duration of the maturation phase, which may take several months in the case of human cells [36]. To achieve this, whole laminin was coated onto the surface of the PAA-ACA gels. It was found that the indirect conjugation of whole laminin using a PLL intermediate was optimal for mNPC-derived neuron attachment for 14 days. Alternatively, direct conjugation of whole laminin with carbodiimide cross-linking chemistry facilitated hNPC-derived neuron attachment for up to 28 days. In both cases, neurons presented TUJ1 and MAP2, indicating the ability of the NPCs to differentiate and mature on the platform. The differences in adhesion behaviors between hNPCs and mNPCs is likely due to intrinsic, species-specific responses. It must be noted that an initial supplement of ROCK inhibitor Y-26732 (5 µM) was required for human NPC attachment [37]. Y-26732 is a potent inhibitor of the Rho GTPase effector ROCK, which is required for the formation of focal adhesions and actin stress fibers—critical components of the mechanotransduction machinery [38]. However, as shown by Ishizaki et al. and others, the initial effects of Y-26732 diminish over time and actin function is restored after 24 h from the initial dose or with withdrawal [39,40,41,42]. Therefore, it is presumed that the inhibitory effect of Y-26732 is negligible in this case as only one dose is administered. An additional limitation of this study is the range of hydrogel stiffnesses investigated. All hydrogels possessed a Young’s modulus > 1 kPa, thus do not provide a physiologically relevant representation of brain tissue. Further works are required to evaluate the fabrication of softer PAA-ACA gels with Young’s moduli < 6 kPa using the method employed here. In summary, the PAA-ACA hydrogel with the ECM coatings described here can be used to reliably modulate multiple biophysical cues and ensure stable long-term neuron attachment, thus paving the way for neural mechanobiology studies.

NPCs have the propensity to differentiate into glial (astrocyte and oligodendrocyte) cell types in vitro, presenting a significant hurdle that must be overcome to improve neuronal yield. Lineage commitment is known to be mechanosensitive and has been shown to be influenced by both topography and stiffness. The second aim of this study was to evaluate the combined effect of or interaction between topography dimensions (2 µm–10 µm) and stiffness (6.2–110.5 kPa) (T*S) on the propensity of mNPCs to differentiate into neurons. Neuronal lineage commitment can be assessed based on the ratio of TUJ1 expressing or TUJ1+ cells to GFAP expressing (GFAP+) cells. TUJ1 expression was significantly higher on the softer substrates and on the topographically enhanced gels relative to the blank. Notably, the percentage of GFAP+ cells was found to have a significant relation with only topography. Additionally, alignment along the gratings was observed, which has been linked to neuronal differentiation. Both TUJ1 and GFAP expression were found to be strongly correlated with T*S, but in opposite directions (negative versus positive, respectively). In other words, softer substrates with smaller micrograting dimensions improved TUJ1 expression and, accordingly, neuronal differentiation. The interaction between topography and stiffness was found to elicit a significantly negative effect on TUJ1 expression, recommending that substrates be designed with the interplay between multiple biophysical cues in mind. Except in the case of the softest gel (6.2 kPa), the 2 µm gratings resulted in the highest TUJ1 expression, with significant relations being drawn in all cases. It is interesting to highlight that the highest percentage of TUJ1 cells was found on the 6.2 kPa gel with 5 µm gratings. It is possible that the combination between the soft, more physiologically relevant substrate and intermediate grating depth increases the neuronal differentiation rate as was similarly observed in Chua et al. [7]. In this work, a greater number of TUJ1 expressing cells was found on gratings with a depth of 4 µm and width and spacing of 2 µm [7]. It is evident that assessing different combinations of topography dimensions and stiffnesses can be utilized to promote commitment to the neuronal lineage over the glial lineage in the absence of biochemical cues.

Neuronal maturation on biomaterials is of great importance as the majority of iPSC-derived somatic cells resemble fetal or neonatal cells [24]. Therefore, neuronal maturation on different topography and stiffness combination was evaluated using the marker MAP2 and morphological characteristics, namely the number of neurite branches and the neurite length. Similar to TUJ1, MAP2 expression was upregulated on the hydrogels compared to the glass coverslip control and was higher on the micropatterned samples relative to the blanks. After the 14-day differentiation and maturation period, the mNPC-derived neurons possessed a neuronal morphology with MAP2+ neurites. The proportion of MAP2+ cells showed a moderate negative correlation with stiffness and a strong negative correlation with topography. However, no significant relations could be drawn between T*S and the percentage of MAP2+ cells could be drawn. Notably, except for the case of the softest gel, no pair-wise comparisons between topography dimensions could be drawn within stiffness groups. These results suggest that mechanosensitive responses associated with neuronal maturation are unique from those involved in differentiation. While topography, stiffness and their interaction were all significantly related to neurite branching, only T*S had a significant relationship with neurite length. Interestingly, the highest percentage of MAP2+ cells, longest neurite, and greatest number of neurite branching was found on the 6.2 kPa gel with the 5 µm gratings. Again, an intermediate grating size on the softest substrate promoted the greatest rate of neuronal maturation. With regards to the hNPC-derived neurons, the results are inconclusive. However, a similar trend, while not statistically significant, can be observed. MAP2 expression is higher on the gels compared to the glass coverslip control and is higher on the topographically enhanced gels compared to the blank controls in both stiffness groups. Within the 110.5 kPa group, the aforementioned trend is observed; the 2 µm gratings enhance MAP2 expression compared to the blank control and increasing the topography dimensions decreases MAP2 expression. Topography does not appear to greatly modulate MAP2 expression on the 6.2 kPa gels. Further investigation is necessary to clarify the combined effects of topography and stiffness on hNPCs.

The results presented here demonstrate that the effects of topography and stiffness cannot be imparted and have the potential to be combined to enhance neuronal differentiation and maturation. While the underlying mechanisms that mediate this response to multiple biophysical cues remains unknown, the geometrical aspects of the gratings, specifically width and depth spacing, and their relationship with stiffness, can be assessed individually to propose a potential explanation. Firstly, as investigated by Zeng et al., the grating width plays a critical role in mediating membrane bending and curvature [43]. In this work, the authors showed that the basal membrane bends into the groove of the grating when the width is greater than 1.9 µm [43]. However, the model developed by Zeng et al. assumes the gratings are not deformable by the cells due to their stiffness. The same cannot be assumed for the gratings here as their softness likely permits deformation by the cells. Therefore, membrane bending into the grating will be modulated by the deformability of the grating stiffness, which will likely influence cytoskeletal organization and contractility, nuclear membrane morphology, and ultimately gene expression. The influence of grating stiffness on stem cell morphology was further explored in Wong et al. [44]. Here, the authors showed that anisotropic rigidity on gratings as a driving force behind cell elongation. Cell elongation and alignment is crucial for polarization and adoption of neural identity [45]. Regarding nuclear morphology, topography-driven neuronal differentiation involves initial changes in nuclear lamin A/C expression [46], a key component of the inner nuclear membrane. The second geometrical feature of relevance is the depth of the gratings. As briefly described previously, the depth of the gratings has been shown to influence neuronal differentiation and neurite outgrowth. The authors propose a neuronal depth-sensing mechanism that is contingent upon the energetic cost of neurite cytoskeleton bending and growth cone dynamics—a process that will be influenced by the substrate deformability [47]. To further address these speculations, it is recommended to investigate the combined effects of topography and stiffness on focal adhesion formation and focal adhesion kinase (FAK) signaling, which are key in mediating NPC cytoskeletal reorganization in response to both stiffness [48,49] and topography [50,51]. Additionally, the Rho family of small GTPases are too worthy of further study due to their role in mediating neuronal differentiation in response to changes in cytoskeletal contractility [52]. Mechanistic studies will provide further insight into how cells respond to numerous cues simultaneously and will facilitate more precise combinations of stiffness and topography for control over cell behavior.

## Data Availability

The data presented in this study are available in the Supplementary Material.

## Supplementary Materials

The following supporting information can be downloaded at: https://www.mdpi.com/article/10.3390/cells12060934/s1, Table S1, The composition of the polyacrylamide (PAA) and N-acryloyl-6-aminocaproic acid (ACA) copolymer (PAA-ACA) hydrogels with varying stiffnesses; Table S2, The composition of the mouse neural progenitor cell (mNPC) maintenance, induction, and maturation media; Table S3, The composition of the human neural progenitor cell (hNPC) maintenance, induction, and maturation media; Table S4, The average percentage of cells β-tubulin III (TUJ1+), glial acidic fibrillary protein (GFAP+) and microtubule associated protein-2 (MAP2+) on PAA-ACA gels of varying stiffnesses and topographies; Table S5, Microtubule-associated protein-2 positive (MAP2+) neurite length and MAP2+ branches per cell on PAA-ACA gels of varying stiffnesses and topographies.
