# Supplementary material for: The Combined Effects of Topography and Stiffness on Neuronal Differentiation and Maturation Using a Hydrogel Platform
Source: Cells. 2023 Mar 18;12(6):934. doi: 10.3390/cells12060934 (PMC10047827; doi:10.3390/cells12060934)
Supplement: Supplementary file 1 [file cells-12-00934-s001.zip › cells-2209237-supplementary.pdf]

Supplementary Information

Table S1: The composition of the polyacrylamide (PAA) and N-acryloyl-6-aminocaproic acid (ACA) copolymer (PAA-ACA) hydrogels with varying stiffnesses.

Reagent	Desired Stiffness			
	Very Soft (6.1 kPa)	Medium Soft (12.9 kPa)	Medium Stiff (22.9 kPa)	Very Stiff (92.6 kPa)
Acrylamide (w/v)	3.0%	4.0%	5.5%	10.0%
Bisacrylamide (w/v)	0.13%	0.17%	0.23%	0.43%
N-acryloyl-6-aminocaproic acid (ACA) (w/v)	1.85%	1.85%	1.85%	1.85%
Tetramethyl ethylenediamine (TEMED) (w/v)	0.1%	0.1%	0.1%	0.1%
Ammonium persulfate (APS) (v/v)	0.1%	0.1%	0.1%	0.1%

Table S2: The composition of the mouse neural progenitor cell (mNPC) maintenance, induction, and maturation media.

Reagent	Volume (out of 50 mL) & Final Concentration		
	Maintenance Media	Induction Media	Maturation Media
DMEM-F12 medium	49 ml	47.5 ml	24 ml
Neurobasal medium	--	--	24ml
Glutamax (100X)	--	0.5 ml (1X)	0.5 ml (1X)
Penicillin/streptomycin (100X)	0.5 ml (1X)	0.5 ml (1X)	0.5 ml (1X)
N2 supplement (100X)	0.5 ml (1X)	0.5 ml (1X)	0.125 ml (0.25X)
B27 supplement (50X)	--	1 ml (1X)	1 ml (1X)
Daily supplement	EGF (20 ng/ml)<br>FGF (20 ng/ml)	FGF (5 ng/ml)	--

Table S3: The composition of the human neural progenitor cell (hNPC) maintenance and differentiation media.

Reagent:	Volume (out of 50 mL) & Final Concentration	
	Maintenance Media	Differentiation Media
DMEM-F12 medium	23.5 ml	24 ml
Neurobasal medium	23.5 ml	24 ml
N2 supplement (100X)	0.5 ml (1X)	0.5 ml (1X)
B27 supplement (50X)	1 ml (1X)	1 ml (1X)
L-glutamine (100X)	0.5 ml (1X)	0.5 ml (1X)
Penicillin/streptomycin (100X)	0.5 ml (1X)	0.5 ml (1X)
Bovine serum albumin (5 mg/ml)	50 µl (5 µg/ml)	--
Human LIF (10 µg/ml)	50 µl (10 ng/ml)	--
CHIR99021 (0.8 mM)	187.5 µl (3 µM)	--
SB431542 (10 mM)	10 µl (2 µM)	--

Table S4: The average percentage of cells β-tubulin III (TUJ1+), glial acidic fibrillary protein (GFAP+) and microtubule associated protein-2 (MAP2+) on PAA-ACA gels of varying stiffnesses and topographies.

**Biophysical Parameters**		**Average percentage of cells expressing neuronal or glial markers**		
Stiffness	Topography	TUJ1+	GFAP+	MAP2+
6.1 kPa	2μmG	26.1%	15.2%	16.9%
	5μmG	36.6%	11.5%	22.9%
	10μmG	27.7%	19.1%	14.5%
	Blank	19.6%	17.0%	7.4%
12.9 kPa	2μmG	35.6%	17.1%	21.1%
	5μmG	27.6%	13.0%	18.3%
	10μmG	22.2%	24.1%	17.6%
	Blank	16.4%	21.0%	11.8%
22.9 kPa	2μmG	29.1%	9.4%	11.8%
	5μmG	21.5%	11.7%	13.2%
	10μmG	17.3%	27.3%	9.13%
	Blank	13.9%	15.2%	11.0%
92.6 kPa	2μmG	24.9%	10.0%	11.6%
	5μmG	22.7%	14.1%	11.9%
	10μmG	16.0%	17.6%	12.8%
	Blank	13.8%	25.1%	9.0%
Glass coverslip (control)		8.4%	25.6%	6.9%

Table S5: Microtubule-associated protein-2 positive (MAP2+) neurite length and MAP2+ branches per cell on PAA-ACA gels of varying stiffnesses and topographies.

**Biophysical Parameters**		**MAP2+ Neurite Length and Branches**	
Stiffness	Topography	Average Neurite Length (µm)	Average Number of Branches
6.1 kPa	2µmG	68.9 ± 8.0	4.1 ± 0.5
	5µmG	80.0 ± 7.5	5.0 ± 0.5
	10µmG	57.9 ± 5.2	3.4 ± 0.4
	Blank	67.3 ± 8.3	4.8 ± 0.7
12.9 kPa	2µmG	68.2 ± 6.5	4.0 ± 0.3
	5µmG	70.7 ± 5.7	3.5 ± 0.3
	10µmG	68.8 ± 6.1	3.2 ± 0.3
	Blank	60.9 ± 4.9	3.5 ± 0.3
22.9 kPa	2µmG	66.2 ± 6.6	3.9 ± 0.4
	5µmG	61.3 ± 5.5	2.8 ± 0.2
	10µmG	66.1 ± 5.8	3.6 ± 0.4
	Blank	62.7 ± 4.2	3.7 ± 0.3
92.6 kPa	2µmG	64.4 ± 6.8	3.8 ± 0.5
	5µmG	61.8 ± 7.5	4.0 ± 0.7
	10µmG	66.7 ± 4.6	4.2 ± 0.3
	Blank	59.0 ± 7.2	4.4 ± 0.5
Glass coverslip (control)		77.9 ± 12.1	5.1 ± 0.7
